# Effect of apelin on cardiac contractility in acute reno-vascular hypertension: The role of apelin receptor and kappa opioid receptor heterodimerization

**DOI:** 10.22038/IJBMS.2018.31361.7555

**Published:** 2018-12

**Authors:** Mahboobeh Yeganeh-Hajahmadi, Hamid Najafipour, Farzaneh Farzaneh, Saeed Esmaeili-Mahani, Siyavash Joukar

**Affiliations:** 1 Physiolgy Research Center, Institute of Neuropharmacology and Department of Physiology and Pharmacology, Kerman University of Medical Sciences, Kerman, Iran; 2 Cardiovascular Research Center, Institute of Basic and Clinical Physiology Sciences and Department of Physiology and Pharmacology, Kerman University of Medical Sciences, Kerman, Iran; 3 Endocrinology and Metabolism Research Center, Institute of Basic and Clinical Physiology Sciences and Department of Physiology and Pharmacology, Kerman University of Medical Sciences, Kerman, Iran; 4 Department of Biology, Shahid Bahonar University of Kerman, Kerman, Iran

**Keywords:** Apelin receptors, Dimerization, Myocardium, Opioid receptors, Reno-vascular hypertension

## Abstract

**Objective(s):**

Apelin/APJ system plays an important role in the regulation of myocardial contractility (MC) and blood pressure. Opioid receptors (OPRs) are also important cardiovascular regulators and exert many of their effects through modulating the function of other systems. This study analyzed the interaction between APJ and kappa OPRs (KOR) in cardiac responsiveness to apelin in acute reno-vascular hypertension (2K1C).

**Materials and Methods:**

MC studies were carried out on 2K1C rats. F13A (APJ blocker), Naloxone (OPR inhibitor), nor-Binaltorphiminedihydrochloride (nor-BNI; kappa OPR inhibitor), PTX (Gi path inhibitor) and chelerytrine (protein kinase C; PKC inhibitor) were administered prior to apelin 20 and 40 μg/kg. The phosphorylation of extracellular signal–regulated kinases (ERK1/2) (PERK) was also assessed. Dimerization of APJ and KOR was evaluated by immunoprecipitation.

**Results:**

Both doses of apelin reduced blood pressure. Apelin 40 exerted a negative inotropic effect, which was inhibited by nor-BNI, but apelin 20 showed a positive inotropic effect, which was resistant to this inhibition. Hypertension increased heterodimerization of the APJ and KOR and this was reduced by apelin 20. F13A, naloxone and PTX significantly reduced PERK in apelin 40 group, but F13A, naloxone, and chelerytrine significantly increased PERK in the apelin 20 group.

**Conclusion:**

The lowering effect of apelin 40 on MC and its non-effectiveness on APJ/KOR dimerization, while augmenting the contractility and reducing the dimerization by apelin 20 implies the APJ/KOR-related effects of apelin on the MC under acute reno-vascular hypertension. This may have potential clinical applications as apelin has been introduced as a potential therapeutic agent in heart failure and opioids are being currently used in the treatment of myocardial infarction.

## Introduction

Cardiovascular disease accounts for approximately one third of the total deaths, and hypertension is responsible for at least 45% of deaths from heart disease and 51% of deaths from stroke (1). Primary renal disease is the most common etiology of secondary hypertension (2).Two*-*kidney-one clip hypertension model (2K1C) is one of the experimental models used to assess secondary hypertension, a comparable condition to kidney disease related hypertension in humans (3).

G-protein-coupled receptors (GPCRs) are the most important targets for drug production especially those for the treatment of cardiovascular diseases such as heart failure and hypertension (4). Recently, many studies have focused on heterodimer formation in GPCRs, and it seems that these heterodimerizations are innovative and promising therapeutic targets (5). 

Apelin and its receptor (APJ) play an important role in the regulation of heart function and blood vessels (6, 7). Opioid peptides and their GPCR receptors are also important regulators of the cardiovascular system (8). Opioid agonists decrease the contractility of myocardium (9). The kappa opioid receptor (KOR) is a common opioid receptor (OPR) in the heart and its expression increases in the heart in response of volume overload (10). It has been shown that KOR stimulation weakens myocardial contraction through protein kinase C (PKC) pathway (11). Both KOR and PKC stimulation reduce Ca^++^ transient in cardiomyocytes and this reduction is inhibited by Chelerytrine (PKC inhibitor) (11). APJ and OPRs can form heterodimer and transmit signals through protein Gi/o, which in turn activates extracellular signal–regulated kinases (ERK1/2) and inhibits adenylate cyclase activity (12, 13). Thus there are similarities between the apelin and opioid systems regarding: 1) the type of receptors, 2) their signaling pathways, and 3) the ability of their receptors to create heterodimer. In our previous study on acute 2K1C animals, we showed positive inotropic effect of apelin 20 mg/kg and negative inotropic effect of apelin 40 mg/kg (14) and different signaling pathway of apelin 20 and 40 mg/kg in lowering blood pressure (15). Also we found reduction in the expression of APJ in the heart, aorta and kidneys of acute 2K1C rats (14, 16). On the other hand, in chronic reno-vascular hypertensive rats a reverse relationship was found between inotropic effects of apelin and APJ/KOR heterodimerization (17). Therefore, as reno-vascular hypertension is associated with changes in the endogenous opioid system (18), and opioids are currently used for managing conditions like myocardial infarction (MI) and stroke, the aim of this study was to evaluate the interaction between apelin/APJ and opioid/OPR systems regarding cardiac responses to apelin in an acute reno-vascular hypertension model and heterodimerization of these two receptors in these conditions. In this regard, we blocked Gi and PKC paths and assessed the phosphorylation of intracellular mediator ERK1/2 in the myocardium as probable mechanisms in the mentioned interactions.

## Materials and Methods

***Materials***

Apelin-13 and APJ antagonist (F13A) were obtained from Phoenix Pharmaceuticals Inc., France. Naloxone hydrochloride dihydrate (non-selective opioid receptor antagonist), nor-Binaltorphiminedihydrochloride (nor-BNI, selective KOR antagonist), β-actin antibody, phosphatase and protease inhibitor were obtained from Sigma-Aldrich Co, Germany. PTX (Gi protein inhibitor) and Chelerytrine (PKC inhibitor), KOR antibody, and APJ antibody were purchased from Santacruz Biotechnology Inc., USA. The other materials were SYBR Green master mix (Applied Biosystems, USA), Column Animal Total RNA extraction Kit (Bio basics INC, Canada), cDNA synthesis kit (Takara Bio INC, Japan), Protein G Sepharose 4 Fast Flow (GE Healthcare life sciences, Sweden), and ELISA kit, which were used for measuring phosphorylated and total ERK1/2(RayBiotech, USA).

***Study groups***

**Figure 1:**
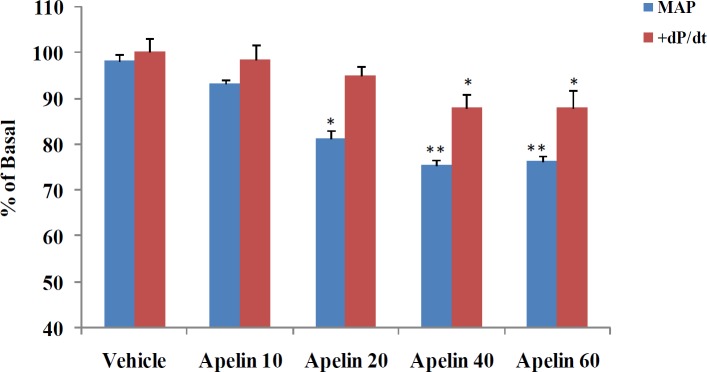
Dose-response data related to the effects of apelin on MAP and + dP/dt max. * = *P*<0.05, ** = *P*<0.01 vs Sham or vehicle. MAP: mean arterial pressure

All experiments were performed according to the national guidelines for animal studies. The study was approved by the ethics committee of the Kerman University of Medical Sciences, Iran (Permission code: 93/189KA). The study included 168 male Wistar rats (180-200 g), which were randomly divided into 21 groups of 8 animals. Two groups of sham and 2K1C were used for assessing the expression of mRNA and protein of KOR and protein of APJ in the left ventricle (see Figure 7 in the result section). Three groups did not receive apelin, including the sham, sham-vehicle and 2K1C-vehicle (as control groups). The remaining 16 groups were divided into two sub-groups of apelin 20 (receiving 20 μg/kg apelin) and apelin 40 (receiving 40 μg/kg apelin). These doses were based on primary experiments related to apelin dose-response curves on cardiac contractility and blood pressure (Figure 1). These experiments showed that the effect of apelin 60 μg/kg on cardiac contractility was similar to apelin 40 μg/kg and exerted no extra lowering effects on the blood pressure. Moreover, as it has been shown that the level of apelin is reduced in 2K1C hypertensive rats (14); in addition, the 20 μg/kg dose would normalize apelin level (physiological dose) and 40 μg/kg would act as a pharmacological dose. Each subgroup consisted of apelin plus 1) saline (vehicle); 2) F13A; 3) naloxone; 4) F13A + naloxone; 5) nor-BNI; 6) F13A + nor-BNI; 7) PTX; and 8) chelerythrine. Apelin, F13A, naloxone, nor-BNI, PTX and Chelerytrine were dissolved in saline.

***Induction of hypertension***

As described previously, hypertension was induced by clipping the left renal artery (14). Concisely, animals were anesthetized with an intra-peritoneal injection of ketamine (80 mg/kg) and xylazine (10 mg/kg). After the incision in the abdominal wall of the flank, a solid Plexiglas clip with a 0.2 mm cleft diameter was placed around the left renal artery. The sham groups were subjected to the same surgery, but no clip was applied. The induction of acute reno-vascular hypertension took four weeks (19).

***Myocardial contractility and blood pressure assessments***

The maximum rate of rise in the left ventricular pressure during systole (+dP/dt max) is often known as an indicator of cardiac contractility and is influenced by preload (here, left ventricular end diastolic pressure, LVEDP) and afterload (here, blood pressure) (20). Also the maximum rate of reduction in pressure during diastole (–dP/dt max) as cardiac relaxation index can be affected by arterial pressure and left ventricular systolic pressure (LVSP) (21). Therefore these variables were measured.

After 4 weeks (19), the animals were anesthetized with intraperitoneal injection of sodium thiopental (50 mg/kg). A polyethylene catheter (PE-50) was placed in the right carotid artery and advanced to the left ventricle. The right femoral artery was also cannulated. The catheters were connected to pressure transducers and were used to measure the left ventricular pressure and contractility indices and the arterial blood pressure, respectively. The left jugular vein was also cannulated for intravenous injection of apelin, APJ antagonist, OPR antagonists or saline. Equal volumes of saline were injected to the sham groups. +dP/dt max, -dP/dt max, LVSP, LVEDP were recorded on an 8-channel Powerlab Physiograph system (AD Instruments, Sydney, Australia). Only clipped rats with a systolic arterial pressure >150 mmHg were included in the study.

After cannulation, at least ten minutes of recovery was allowed to stabilize the pressure. Saline or antagonists (except PTX) were administered ten minutes before the administration of apelin. To wash the drug from the cannula, after administration of each drug, 0.2 ml of saline was injected. The antagonists included F13A (30 μg/kg) (14), naloxone (3 mg/kg) (22), nor-BNI (3 mg/kg) (22) and chelerythrine (5 mg/kg) (23). PTX (10 µg/kg) was injected intra-peritoneally 48 hours prior to the experiments (24).

**Figure 2:**
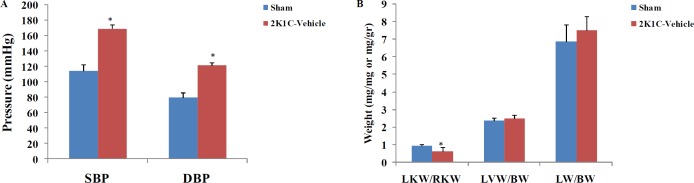
Effects of clamping of the left renal artery on the ratio of the weight of left (LKW) to right kidnys (RKW), left ventricle (LVW) and lungs (LW) to body weight (BW), and on hemodynamic parameters of sham and 2K1C-vehicle groups (n=8 in each group). * = *P*<0.05. SBP: systolic blood pressure; DBP: diastolic blood pressure. The unit of ordinate for LKW/RKW is mg/mg and for LVW/BW and LW/BW is mg/gr

**Figure 3:**
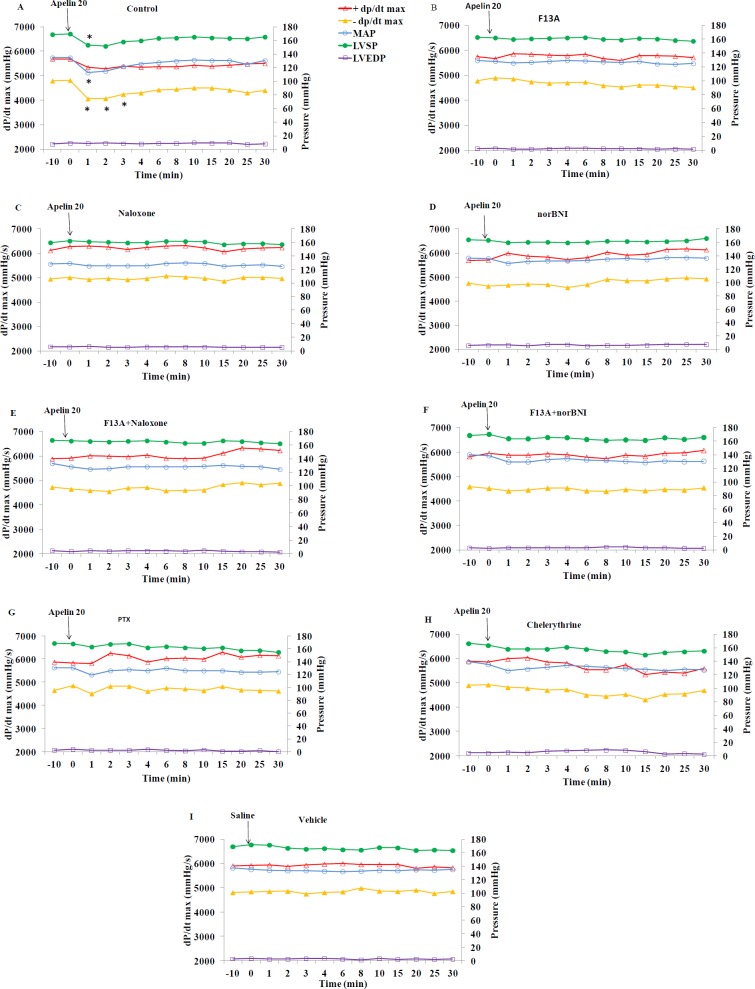
Time course of changes in +dP/dt max, -dP/dt max, MAP, LVSP and LVEDP in apelin 20 μg/kg, after saline (control) or different antagonists/inhibitors (n=8 in each group). Saline or antagonists/inhibitors were injected at min -10 and apelin at dose 20 µg/kg (or saline in panel I) at time 0. MAP: mean arterial pressure, LVSP: left ventricular systolic pressure, LVEDP: left ventricular end diastolic pressure. * = *P*<0.05 vs zero time point

**Figure 4:**
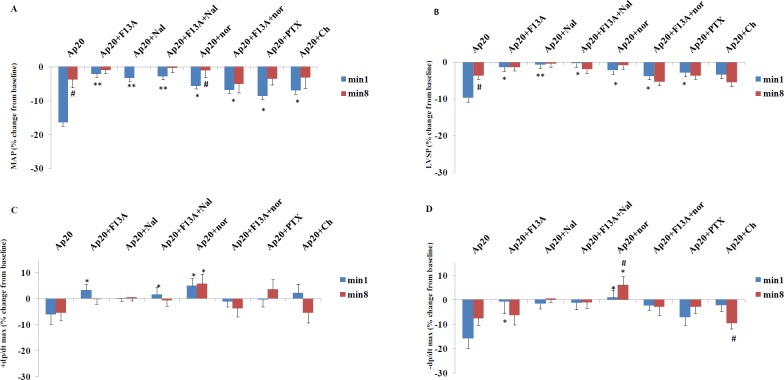
Changes in MAP, LVSP and ±dP/dt max from baseline at min 1 and 8 in response to apelin 20 µg/kg in different groups of the study (n=8 in each group). Saline or antagonists/inhibitors were administered 10 minutes before apelin injection. MAP: mean arterial pressure, LVSP: left ventricular systolic pressure. *=*P*<0.05, **=*P*<0.01 vs. apelin at the same time point. #=*P*<0.05 min 1 vs min 8 of the same group

**Figure 5:**
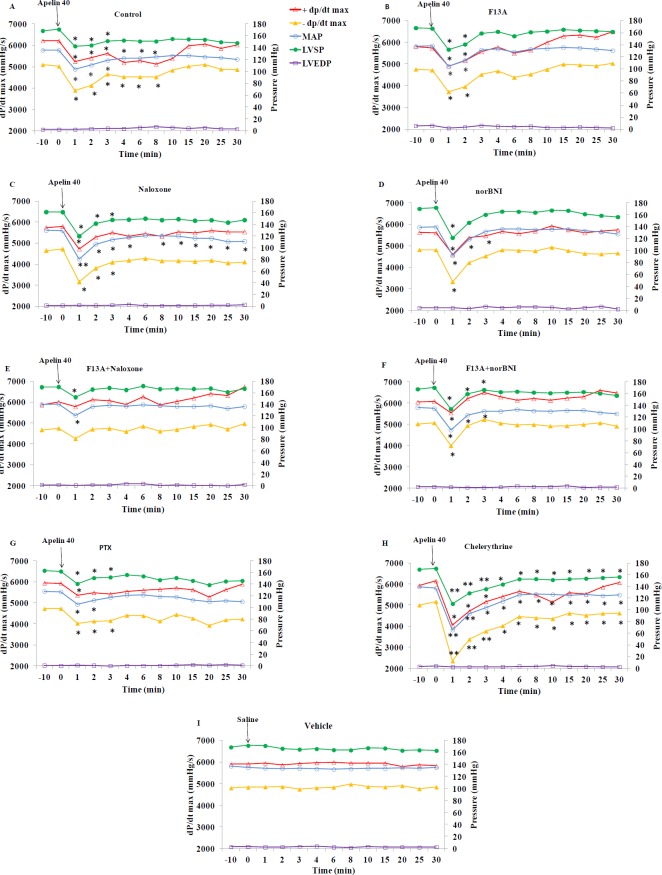
Time course of changes in +dP/dt max, -dP/dt max, MAP, LVSP and LVEDP in apelin 40 μg/kg, after injection of saline (control) or different antagonists/inhibitors (n=8 in each group). Saline or antagonists/inhibitors were injected at min -10 and apelin at dose 40 µg/kg (or saline in panel I) at time 0. MAP: mean arterial pressure, LVSP: left ventricular systolic pressure, LVEDP: left ventricular end diastolic pressure. * = *P*<0.05, **=*P*<0.01 vs. zero time point

**Figure 6:**
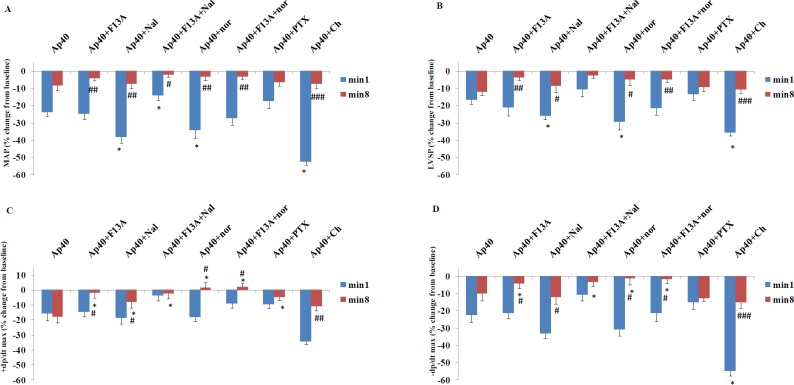
Changes in MAP, LVSP and ±dP/dt max from baseline at min 1 and 8 in response to apelin 40 µg/kg in different groups of the study (n=8 in each group). Saline or antagonists/inhibitors were administered 10 minutes before apelin injection. MAP: mean arterial pressure, LVSP: left ventricular systolic pressure. *=*P*<0.05, **=*P*<0.01 vs. Apelin at the same time point. #=*P*<0.05, ##=*P*<0.01, ###=*P*<0.001 min 1 vs. min 8 of the same group

**Figure 7:**
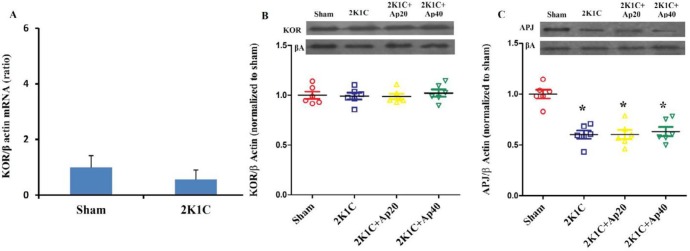
A: Expression of kappa opioid receptor mRNA of sham and 2K1C , B: Expression of kappa opioid receptor protein and C: apelin receptor protein, in the left ventricle of sham, 2K1C, 2K1C + apelin 20 and 2K1C + apelin 40 groups (n= 8 in each group)

**Figure 8:**
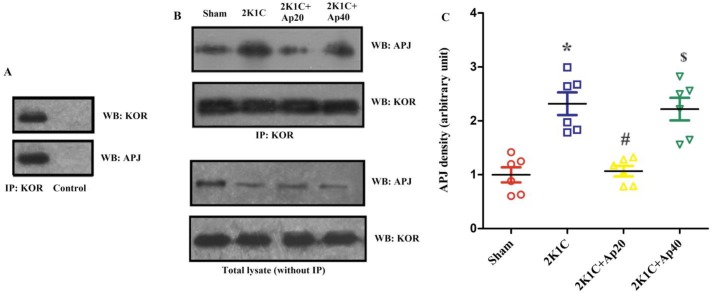
Heterodimerization of APJ and KOR in the left ventricle of 2K1C animals and the effect of apelin 20 and 40 µg/kg on it. A: Co-immunoprecipitation with KOR and control antibodies. B: Western blotting was performed for APJ or for KOR after KOR was immunoprecipitated (upper panel) or for total lysate without immunoprecipitation (lower panel). C: Quantification of the data in B (upper panel) (n=6 in each group). Ap20: Apelin 20 µg/kg , Ap40: Apelin 40 µg/kg * = *P*<0.05 vs. Sham. # = *P*<0.05 vs 2K1C. $ = *P*<0.05 vs. 2K1C + Ap20

**Figure 9:**
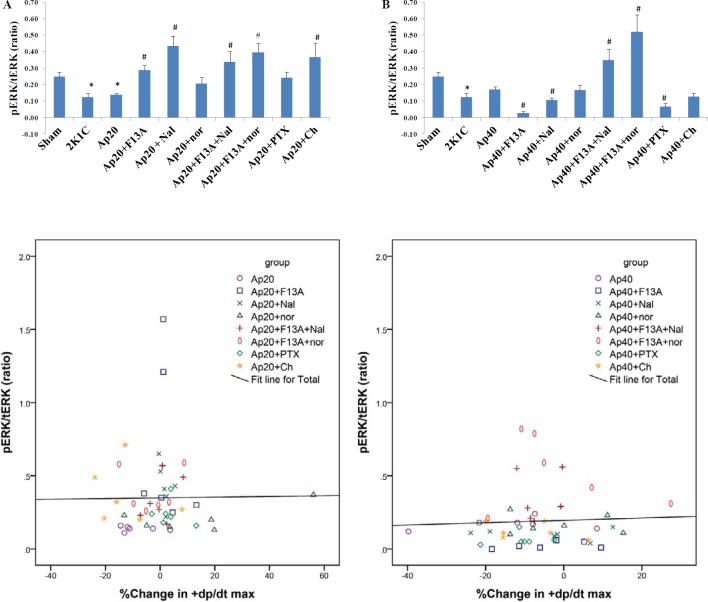
PERK /tERK ratio in the groups receiving apelin 20 (A) or apelin 40 (B). Scatterplot with fitted regression lines showing the relationship between % change in +dp/dt max and PERK 1/2 in apelin 20 groups (C) and apelin 40 groups (D). n=6 for each group. PERK: phosphorylated extracellular signal–regulated kinases, tERK: total ERK. Ap20: Apelin 20 µg/kg, Ap40: Apelin 40 µg/kg, Nal: Naloxone, nor: nor-BNI, Ch: Chelerythrine. * = *P*<0.05 vs. Sham, # = *P*<0.05 vs apelin 20 or apelin 40 µg/kg

**Figure 10:**
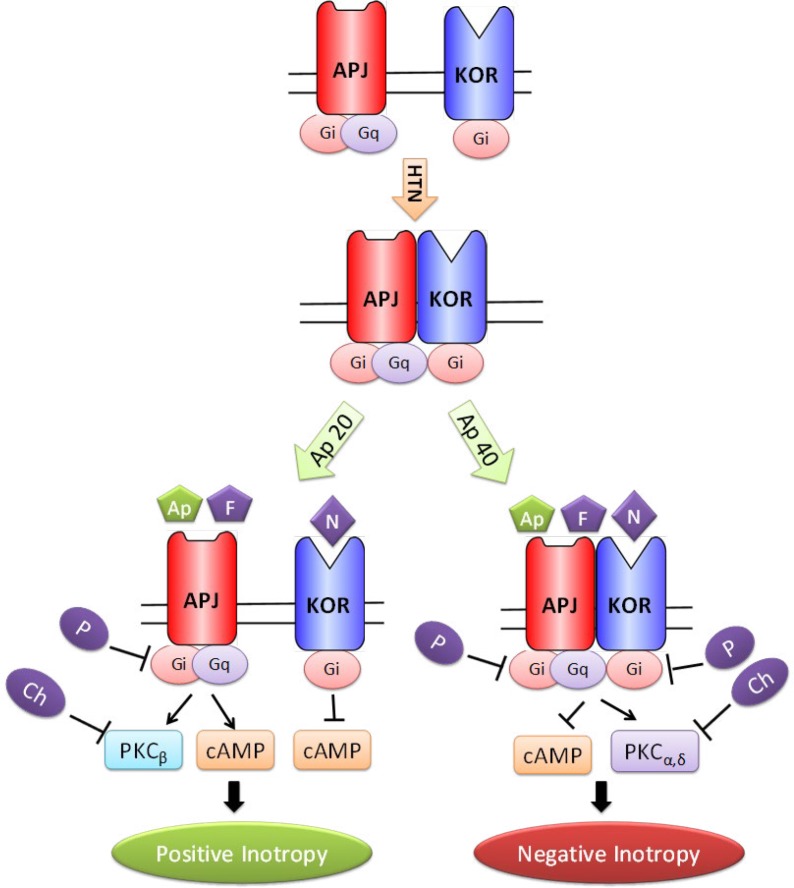
Schematic presentation of possible mechanisms of positive and negative inotropic effect of apelin 20 and apelin 40 in acute renovascular hypertension. Ap: apelin, APJ: apelin receptor, Ch: chelerytrine, F: F13A, HTN: hypertension, KOR: kappa opioid receptor, N: nor-BNI, P: PTX

At the end of the experiment, the animal was euthanized in deep anesthesia. Immediately, the heart, lungs and kidneys were removed, washed in saline and dehumidified using a sterilized cloth. The right ventricle and atria of the heart were removed using scissors, and the left ventricle (plus septum), lungs and kidneys were weighed. The weight ratio of left kidney to the right kidney was used as the kidney ischemia index, and the ratio of lung weight to the body weight was considered as the pulmonary edema index. The ratio of left ventricular weight to body weight is the left ventricular hypertrophy index. The free wall of the left ventricle was quickly immersed in liquid nitrogen and stored in -80 ^°^C for later molecular experiments.

***mRNA and receptor protein expression analysis***

KOR and APJ protein levels in the heart of sham, 2K1C, 2K1C + apelin 20 and 2K1C + apelin 40 groups were evaluated using western blotting test (25). Relative expression levels of KOR mRNA were analyzed using Step One Plus real time PCR system (Applied Biosystems Corporation, Foster city, California, USA). From each sample of left ventricle, 35 mg was taken and homogenized. The total RNA was extracted according to the RNA kit protocol. With 500 ng of RNA, cDNA synthesis was performed using Takara synthesis kit based on the kit protocol and with the final volume of 10 µl. RT-qPCR was performed using SYBR Green Master mix, 2 µl of cDNA, 0.8 µl of each of the primers (26) (concentration of 10 µM) at the final volume of 25 µl.

The temperature conditions were as follows: initial denaturation step at 95 ^°^C for 15 minutes, 40 cycles of denaturation at 95 ^°^C for 20 seconds and annealing at 59 ^°^C for 50 seconds. mRNA and protein expression levels were normalized in relation to beta actin using CT and 2 ^–ΔΔCT ^method (27).

***Immunoprecipitation***

Immunoprecipitation was used for assessing dimerization of APJ and KOR. For this purpose, 20 mg of the left ventricle tissue was homogenized in 4 ml of TNE solution containing protease inhibitor. Then, it was centrifuged at a speed of 15000 rpm at 4 ^°^C for 15 min. In the first step to precipitate the interested proteins, 50 μl of the supernatant was mixed with TNE to reach a volume of 194 μl, and then 6 μl of KOR antibody or control antibody (negative control) was added. The mixture was incubated at 4 ^°^C overnight on end-over-end shaker. The Protein G Sepharose beads (GE Healthcare) were added and incubated under the same conditions for three hours. In the second step to evaluate heterodimerization, western blotting was performed for proteins. For this purpose, the precipitated proteins and beads were washed 5 times by TNE. After that to perform elution, 50 μl of 2X buffer sample was added for 5 minutes at 95 ^°^C. Then, western blotting was performed for APJ or KOR using 20 µl of each sample (28). Western blotting was also performed for total lysate without immunoprecipitation as positive control.

***Measuring ERK1/2 phosphorylation***

ERK1/2 phosphorylation was measured by an ELISA kit. In brief, approximately 15 mg of the left ventricle tissue was homogenized in 500 μl of lysis solution containing phosphatase and protease inhibitor. Then, it was centrifuged for 15 minutes at 15000 rpm and 4 ^°^C. The extracted protein was diluted with assay diluent solution. The rest of the process was carried out according to the protocol of the kit. Finally, after reading the light absorption of the samples, blank light absorption was subtracted from the light absorption of each sample, and the ratio of light absorption of phosphorylated ERK to total ERK was calculated.

***Statistical analysis***

Data are presented in the Figures as the means ± SEM. To compare the effect of apelin on hemodynamic and contractility parameters in the presence and absence of antagonists over time, two way repeated measure ANOVA was used. The first factor was treatment groups (8 groups) and the second factor was time as repeated measure. Significant differences between treatment and time were evaluated by Bonferroni *post hoc* test. For western blot, RT-PCR and ERK1/2 variables, differences among groups were analyzed by one way ANOVA followed by Tukey’s *post hoc* test. Student paired t-test was used to compare between minutes 1 and 8 of hemodynamic variables in each group. Pearson’s correlation coefficient was performed to evaluate correlation between heart contractility and ERK1/2 phosphorylation. *P*<0.05 was considered to be significant.

## Results

***The effect of renal artery clamping***

The effects of left renal artery clamping on systolic and diastolic blood pressure, the ratios of left kidney weight to the right kidney weight, left ventricle weight to the body weight and lung weight to the body weight are shown in the Figure 2A and 2B. Clamping led to significant increase in the systolic and diastolic blood pressure and significant decrease in the ratio of left kidney weight to the right kidney weight.

***The effects of apelin and antagonists on cardiac contractility***

Figure 3 shows the time course of changes in ±dP/dt max and factors affecting them, i.e. LVEDP, LVSP, and mean arterial pressure (MAP) in response to apelin 20 in the presence of saline or various antagonists. The quantity of these changes in minutes 1 and 8 are shown in Figure 4. Apelin 20 caused significant drops in MAP and LVSP during the first minute (from 134.6±1.3 to 112.5±6.9 mmHg and from 169.7±2.5 to 153.2±4.0 mmHg, respectively) (*P*<0.05) (Figure 3A). +dP/dt max showed a tendency to decrease, but it was not significant. -dP/dt max reduced significantly during the first 3 minutes and did not recover completely in ten minutes (*P* <0.05) (Figure 3A).

F13A, naloxone, and the combination of naloxone and F13A inhibited the changes of MAP, LVSP, +dP/dt max and -dP/dt max in response to apelin 20 (Figure 3B-E and Figure 4A-D). Nor-BNI partially inhibited the drop in MAP caused by apelin 20. In the presence of nor-BNI, +dP/dt max showed an increasing tendency (Figure 3D and 4C). Changes in MAP and +dP/dt max in response to apelin 20 were not significant in the presence of nor-BNI + F13A, and the tendency to increase in +dP/dt max, which was observed in the presence of nor-BNI alone, was lost (Figures 3F and 4C).

There were no changes in hemodynamic parameters in 2K1C-vehicle group (Figures 3I and 5I) in response to saline injection. The data for sham and sham-vehicle groups were not included in the Figures as they were normotensive groups (methodology controls), and did not concern this study.

Figure 5 shows the time course of changes in MAP, LVSP, LVEDP and ±dP/dt max in response to apelin 40 in the presence of saline or different antagonists. The quantity of these changes in minutes 1 and 8 are also shown in Figure 6. Apelin 40 caused significant drops in MAP and LVSP during the first three minutes (*P<*0.05) (Figure 5A). +dP/dt max and -dP/dt max also fell significantly, which lasted for 8 minutes (*P<*0.05).

F13A could not inhibit the drop in MAP and LVSP in response to apelin 40, but it inhibited the drop in +dP/dt max and -dP/dt max after the third minute completely (Figures 5B and 6).Naloxone exacerbated the drop in MAP in response to apelin 40 (from 129.5±1.5 to 80.7± 6.7 mmHg) (*P* <0.01), and this reduction continued to the end of the study (*P<*0.05). However, the drop in +dP/dt max was significant only in the first minute (*P<*0.05), and from the second minute onwards it returned to the basal level. The drop in -dP/dt max and LVSP in the presence of naloxone was significant for 3 minutes (Figure 5C). In the presence of nor-BNI, the drop in MAP was significant for 3 minutes and the drop in LVSP, +dP/dt max and -dP/dt max was significant only for the first minute (*P* <0.05) (Figures 5D and 6).

The MAP and LVSP drops due to apelin 40 in the simultaneous presence of F13A and naloxone were partially inhibited (16% vs. 28%) (*P* <0.05), yet drops in +dP/dt max and -dP/dt max were inhibited almost completely (Figures 5E and 6C-D).

The MAP and LVSP drops in response to apelin 40 in the simultaneous presence of F13A and nor-BNI were not inhibited in first 3 minutes. Decreases of +dP/dt max and -dP/dt max were not also affected during the first minute (Figure 5F and 6C-D).

Gi inhibition by PTX could not inhibit the drop in the MAP and LVSP in response to apelin 40 completely, but it prevented the drop of +dP/dt max from the third minute and the drop in -dP/dt max (Figure 5G and 6). Chelerythrine intensified the drop in MAP and LVSP in response to apelin 40 (*P* <0.01), which did not return to its basal level until the end of the study. The drop in +dP/dt max after the fourth minute was inhibited significantly in the presence of Chelerythrine, but the drop in -dP/dt max remained significant until the end of study (Figures 5H and 6C-D). Changes observed in the LVEDP during the study were not significant in response to apelin 20 or 40 µg/kg alone or in the presence of different antagonists/inhibitors.

***Western blotting and qPCR***

KOR mRNA and protein expression levels are shown in Figure 7. Reno-vascular hypertension had no significant effect on the expression of KOR mRNA and protein (Figure 7A-B). ). Apelin 20 and apelin 40 did not affect the KOR protein levels in the left ventricles of hypertensive rats (Figure 7B). APJ protein expression reduced in the left ventricles of 2K1C, 2K1C + apelin 20 and 2K1C + apelin 40 groups (Figure 7C).

***Immunoprecipitation***

Immunoprecipitation of KOR followed by western blotting of APJ showed that dimerization of the two receptors exists in normotensive rats (Sham). Reno-vascular hypertension increased heterodimerization by 2.3 times (*P*<0.05). Apelin 20 recovered the heterodimerization to the level of the sham (*P*<0.05), but apelin 40 failed to recover the heterodimerization of the two receptors (Figure 8).

***ERK phosphorylation***

The ERK1/2 phosphorylation in 2K1C rats was significantly lower compared to the Sham. Apelin 20 did not cause any significant changes in the phosphorylation of ERK, but the addition of F13A, naloxone, F13A + naloxone, F13A + nor-BNI and chelerythrin antagonists to apelin 20 significantly increased ERK1/2 phosphorylation. Yet, the addition of PTX and nor-BNI alone did not change the phosphorylation of ERK1/2 (Figure 9A).

Apelin 40 did not cause significant change in the phosphorylation of ERK1/2. F13A, naloxone and PTX significantly reduced the phosphorylation of ERK1/2. F13A + naloxone and F13A + nor-BNI significantly increased the phosphorylation of ERK1/2 (Figure 9B). Figures 9 C and D show the scatter plots for the data and correlation lines between ERK1/2 phosphorylation and contractility changes. Despite the changes in phosphorylation of ERK1/2 by some interventions, correlation lines show that there was no correlation between the level of phosphorylated ERK1/2 and +dp/dt max in the studied groups.

## Discussion

In this study, the cardiac effects of two different doses of apelin and the interaction of APJ with OPR in the heart of reno-vascular hypertensive rats were investigated. 

+dP/dt max that is often known as an indicator of cardiac contractility is influenced by preload and afterload (20). Therefore, when this variable is investigated as an indicator of cardiac contractility, changes of the two other factors should also be taken into consideration. In this study, apelin 20 and 40 µg/kg did not cause any significant changes in LVEDP (preload index). Apelin 20 significantly reduced the MAP **(**afterload index) by 16%, but it did not cause a significant reduction in the +dP/dt max. This shows the positive inotropic effect of apelin 20. According to Frank Starling mechanism, it was anticipated that cardiac contractility decreases in accordance with the drop in the afterload (arterial pressure). Therefore, the increase in cardiac contractility has masked the lowering effect of the drop in MAP on +dP/dt max. 

Nor-BNI partially inhibited the MAP drop caused by apelin 20. However, in the presence of the 6% drop in MAP, +dP/dt max showed a tendency to increase. Therefore, it seems that KOR inhibition can augment the positive inotropic effect of apelin 20. The simultaneous administration of F13A and nor-BNI significantly inhibited the drop in MAP and increased +dP/dt max in the nor-BNI group, which shows that both the vascular and cardiac effects of apelin 20 are induced through APJ as these effects are also inhibited by F13A.

Apelin 40 decreased MAP in the first 3 minutes after injection. +dP/dt max significantly declined after the injection of apelin 40, which lasted for 8 minutes. Also, despite gradual recovery of MAP, +dP/dt max continued its drop and it was not influenced by the changes in MAP. This reflects the negative inotropic effects of apelin 40, especially from the third to eighth minutes. The study of Perjes *et al.* on isolated rat heart showed that the effect of apelin on cardiac contractility appears at least 3 minutes after its administration (29). Therefore, changes in contractility index observed in the first three minutes in the present study are consequences of the alterations in the afterload (blood pressure) and the subsequent changes are direct responses of the heart to apelin.

F13A failed to inhibit the drop in MAP caused by apelin 40; thus, the vascular effects of apelin 40 are not induced through APJ. Yet, changes in +dP/dt max were in comply with MAP, as in the presence of F13A, and both MAP and +dP/dt max return to the basal level. Therefore, it seems that the APJ inhibition by F13A could inhibit negative inotropic (cardiac) effects of apelin. In other words, unlike the vascular effects, the cardiac inotropic effects of apelin 40 are induced by APJ.

Naloxone exacerbated the effects of apelin 40 on arterial pressure (from 25% to about 37% drop), which did not fully return to the pre-apelin injection level by the end of the study. Thus, OPRs oppose the dilator effects of apelin 40 that were induced independent from the APJ. However, despite this sharp drop in MAP, +dP/dt max returned towards the basal level. In the presence of naloxone, apelin 40 caused a small and significant drop in +dP/dt max only in the first minute, which was related to reduction in blood pressure. Thus, the cardiac effects of apelin 40 induced by APJ ) 3 min after injection) were mediated by OPRs. As a result, OPRs participate in the negative inotropic effects of apelin 40. This was in contrary to the positive inotropic effects of apelin 20. In this regard, the effects of nor-BNI were similar to the effects of naloxone. Therefore, it seems that KORs mediate the negative inotropic effects of apelin 40 and other OPRs have no role in this process. The use of a combination of F13A along with two other antagonists (naloxone or nor-BNI) was similar to using nor-BNI alone. This reaffirms that KORs mediate the cardiac effects of apelin 40 and are the downstream to the APJ receptors.

Given that it has been shown that the positive inotropic effects of apelin in the isolated hearts of normotensive rats are dependent on Gi and PKC (29), the effect of inhibiting these pathways on positive and negative inotropic effects of apelin 20 and 40 µg/kg in this model of hypertension were evaluated. *In vivo *Gi inhibition by PTX partially inhibited the blood pressure-lowering effect of apelin 20 and inhibited its inotropic effects. PKC inhibition by chelerythrine significantly inhibited the blood pressure-lowering effect of apelin 20 and its related +dP/dt fall (min 1), but it does not seem to affect its inotropic effects. Figure 3H shows that the use of chelerythrine led to an increase in LVEDP after 3 minutes. This suggests that contractility was also reduced. It seems that PKC plays a role in creating positive inotropic effects of apelin. These results are in agreement with the study of Szokodi *et al.* on isolated hearts, which showed that PKC mediated the positive inotropic effects of apelin (30).

PTX did not prevent the initial drop in MAP caused by apelin 40; yet, it inhibited the negative inotropic effects from minute three onwards. Chelerythrine exacerbated MAP drop caused by apelin 40; however, it also inhibited the negative inotropic effects of apelin 40 after minute three. Consequently, it seems that although the vascular effects of apelin 40 are not inhibited by Gi and PKC suppression, the cardiac effects are inhibited by Gi and PKC suppression. These imply that the two doses of apelin lead to different signaling pathways in the heart (Figure 10), and these signaling pathways are different from those in the vessels. Previous *in vitro* studies have shown that the pairing of G protein and APJ is probably different in VSMC and in the myocardial cells (31).

It has been demonstrated that different concentrations of ligands may affect GPCRs differently. For instance: 1) Pain reduction curve in response to apelin is U-shape and dose dependent. A medium dose of apelin exerts the best effect. At high doses, apelin probably activates a receptor other than the classic receptors that opposes the pain-reducing effect of the optimum dose (32). 2) Different doses of apelin exert their effects through different pathways in the hypothalamus (33). While low dose of apelin increased nitric oxide (NO) production, its high dose led to the production of hydrogen peroxide, which is an endothelium-derived hyperpolarizing factor (EDHF) (34). 3) CGP12177A, a β1 and β2 receptor antagonist, exerts antagonistic effect on β1-adrenergic receptors (β1-ADRs) at low concentrations and agonistic effects at high concentrations (35). Studies on human β1-ADRs suggest that there are binding sites that are able to stimulate functional responses other than those that are attributed to classic orthoseric sites. These binding sites are able to function both independently and cooperatively (36). Consequently, this possibility also holds true for APJ to have binding sites other than its classic sites. These sites may have been activated by high dose of apelin and could act through different signaling pathways.

-dP/dt max that is considered as an indicator of heart relaxation can be affected by arterial pressure and LVSP (21). Thus, when changes in -dP/dt max are investigated, changes of the two other factors should also be taken into account. As previously described, in spite of the return of MAP and LVSP into their pre-apelin levels, the continued drops (statistically significant) in -dP/dt max lasted for 8 minutes. Therefore, it seems that both apelin 20 and 40 exert negative lusitropic effects on the heart of acute reno-vascular hypertensive rats, especially from the third minute. Despite the opposite (negative and positive) effects of apelin 20 and 40 on +dP/dt max, their effects on -dP/dt max were in the same direction. Due to the fact that in spite of intensified pressure drop in response to apelin 40 in nor-BNI group, the drop in-dP/dt max between minutes 3-8 could be inhibited, so it seems that KORs are the mediators of the negative lusitropic effect of apelin. Regarding the lack of inhibition of -dP/dt max by PTX and its intensifying by chelerythrine, it can be concluded that PKC opposed the negative lusitropic effect of apelin 40.

Immunoprecipitation showed that reno-vascular hypertension led to an increase in heterodimerization of APJ and KOR. Apelin 20 significantly reduced the amount of heterodimerization in acute reno-vascular hypertension, but this was not the case with apelin 40. This is in-line with the obtained cardiac findings since apelin 20 had positive inotropic effects and apelin 40 had negative inotropic effects. Previous studies have shown that heterodimerization of GPCRs may lead to a change in the coupling of G-protein-receptor and also changes the type of the coupled G protein, activating different signaling pathways (37-39). It is likely that changes in the amount of heterodimerization of APJ and KOR participate in different signaling pathways of the two doses of apelin mentioned above. In our previous work on chronic 2K1C rats, two doses of apelin exerted positive inotropic responses with reductions in APJ/KOR heterodimerization in both doses (17), showing a reverse relationship between APJ/KOR heterodimerization and inotropy in the heart. 

The reduction of APJ protein expression along with no alteration in the expression of KOR suggests that the increased dimerization of these two receptors cannot be the result of increased density of their proteins in the cell membrane, and may have functional significance, i.e it has happened in the downstream pathways to the receptors. We showed that apelin 20 reduces the KOR and APJ heterodimerization. Cai *et al.* showed that about 60% of APJs in cell membrane are in dimeric or oligomeric form (40) and stimulation by apelin further increased the density of APJs oligomer. Thus, stimulation by apelin 20 may have increased the APJs oligomers and reduced the APJ availability for heterodimeriztion with KOR. As mentioned above, it is possible that there are binding sites other than classic orthoseric sites that may have been affected by different ligand dose (36). Thus, it is possible that apelin 40 by affecting these sites changes the APJs properties and stabilize APJ/KOR heterodimers.

Both the positive and negative inotropic effects of apelin were inhibited by chelerythrine. The reason may be the presence of four different isoforms of PKC in heart, which affect the myocardial contractility differentially. While PKCβ increases the heart contractility, contractility decreases by PKCα and PKCδ (41). 

The results of ERK1/2 phosphorylation assessment showed that antagonists F13A, naloxone and PTX inhibited the inotropic effects of both doses of apelin, but in their presence apelin 20 increased and apelin 40 decreased the level of PERK. As apelin 20 exerted positive and apelin 40 exerted negative inotropic effects, it seems that ERK phosphorylation is associated with changes in inotropic properties of the heart. However, this is not a consistent rule because for example in the presence of nor-BNI and chelerythrine apelin 40 did impose significant change in PERK, while it inhibited and increased the +dP/dt max, respectively. The flat correlation lines between ERK1/2 phosphorylation and contractility changes data supports this implication. Therefore, phosphorylated ERK may have not mediated the inotropic response of apelin in reno-vascular hypertensive rats. In a study by Perjes *et al*., PKC inhibition suppressed the positive inotropic effect of apelin in isolated hearts of normal rats but could not inhibit ERK phosphorylation (29). It has been shown that APJ can couple up with both Gi and Gq and activate different downstream signaling pathways e.g ERK or Akt (42). In a recent study, it was shown that ERK and Akt play opposite roles in the positive inotropic response to steroids (43). Thus, it is possible that in the presence of increased heterodimeriztion of KOR and APJ here, the Akt pathway acts as the dominant pathway.

## Conclusion

The lowering effect of apelin 40 on cardiac contractility along with its non-effectiveness on APJ-KOR dimerization, in addition augmenting the contractility and reducing the dimerization by apelin 20, along with their reverse effects on the level of PERK, implies the dose-dependent differential effects of apelin on the heart in acute reno-vascular hypertension. Since apelin and its receptor APJ are considered as a potential therapeutic target in the treatment of cardiovascular diseases, and opioids are currently used in treating stroke and MI, further research on the interaction of these two systems may offer a new subject to research for therapeutic application of these findings.
